# Cardiovascular Toxicity of Metal-Based Nanoparticles

**DOI:** 10.3390/ijms26125816

**Published:** 2025-06-17

**Authors:** Eun-Hye Kim, Sehyeon Park, Ok-Nam Bae

**Affiliations:** 1 College of Pharmacy, Kyungsung University, Busan 48434, Republic of Korea; dldh615@ks.ac.kr; 2 College of Pharmacy, Institute of Pharmaceutical Science and Technology, Hanyang University, Ansan 15588, Republic of Korea; pshsh6656@naver.com

**Keywords:** nanoparticle, metal-based nanoparticles (MNPs), cardiovascular system, cardiovascular toxicity

## Abstract

The rapid development of nanotechnology has led to increased human exposure to metal-based nanoparticles (MNPs) through inhalation, ingestion, and dermal contact, raising growing concerns on their potential health effects. Due to their nanoscale size and unique physicochemical properties, the MNPs can translocate from the initial exposure sites to the circulatory system and accumulate in the body. This review focuses on MNP-induced cardiovascular toxicity, highlighting its biodistribution, cytotoxic mechanisms, and pathological impact associated with various cardiovascular diseases. MNPs disrupt endothelial function, promote oxidative stress, and induce apoptosis and ferroptosis in cardiovascular cells. Furthermore, MNPs increase endothelial permeability, impair blood–brain barrier integrity, and enhance procoagulant activity, thereby contributing to vascular and cardiac dysfunction. The particles and their released metal ions play a synergistic role in mediating these toxic effects. Here, we focused on the effects of nano-sized particles while incorporating recent in vitro and in vivo studies that address the cardiovascular impacts and mechanisms of MNP-induced toxicity. This comprehensive review will help understand and explain the potentially toxic effects of MNPs on the cardiovascular system.

## 1. Introduction

Recent developments in nanotechnology have led to the emergence of nanomaterials that are utilized in various fields, including research, industry, and medicine [1,2,3]. Therefore, people are easily exposed to various nanomaterials through nanomedicine and consumer goods, including cosmetics, sunscreen, pharmaceuticals, food additives, and paints [4]. The use of consumer goods containing nanomaterials is closely linked to human exposure to nanoparticles. In the United States, the amounts of discharged nanoparticles from consumer goods were at least 2.67–3.1 × 10^3^ metric tons/year into landfills and the environment in 2014, equivalent to about 9.42 g/person per year. Among them, 36–43%, 0.7–0.8%, 28–32%, and 24–36% were identified in landfills, air, water bodies, and soil systems, respectively [5]. Exposure to nanomaterials occurs through ingestion, inhalation, and direct contact [6]. Due to their nanosize, they can translocate to the circulatory system from primary target organs, including the gastrointestinal tract, lungs, and skin [7,8]. Circulating nanoparticles either move to secondary target organs, such as the kidney, heart, and brain, or continuously circulate in the bloodstream. Magnetite nanoparticles were detected in brain tissue, indicating that they can be transported through the olfactory bulb or the blood–brain barrier (BBB) and in the bloodstream of healthy humans [9]. These findings suggest that circulating nanoparticles can affect cardiovascular components, including endothelial and blood cells. The impaired cardiovascular system is closely linked with the development of cardiovascular diseases, which are significant causes of morbidity and mortality worldwide [10,11]. Previous reviews have focused on the contribution of nanomaterials to the toxicity and pathological alterations of cells and tissues in exposed organs. Our review suggests that nanomaterial-induced toxicity is associated with cardiovascular damage and disease.

Metal-based nanoparticles (MNPs) are produced from metals by destructive or constructive methods [12]. MNPs are majorly derived from aluminum, lead, silver, gold, iron, cobalt, zinc, titanium, silica, cadmium, and copper, which are widely used in various applications (Table 1) [13]. As the application of MNPs grows, regulatory perspectives and safety guidelines are also evolving. In the European Union (EU), MNPs are subject to specific provisions under the REACH and CLP regulations for cosmetics and food, which require separate registration and safety data for nanoforms [14]. Also, the European Food Safety Authority (EFSA) provides technical guidelines for evaluating nanoparticle exposure and toxicity [15]. In the United States, the Environmental Protection Agency (EPA) regulates nanomaterials under the Toxic Substances Control Act (TSCA), while the Food and Drug Administration (FDA) and the Occupational Safety and Health Administration (OSHA) oversee their use in food, drugs, and workplace safety [16,17].

MNPs have unique characteristics due to their size, which ranges from 10 nm to 100 nm. MNPs can be classified into two categories: metal and metal oxide nanoparticles [18]. Investigations of miners and refinery workers exposed to MNPs and having pulmonary disease have demonstrated a correlation between exposure to MNPs and disease incidence [19,20,21]. MNPs induce various toxicity via various mechanisms, including oxidative stress, inflammation, and cell death (Table 1) [22,23,24,25]. MNPs can release metal ions from particles, and it is essential to understand the mechanisms of MNP-induced toxicity due to the released metal ions. In this review, we focused on investigating whether MNP-induced toxic mechanisms involve particles themselves, ions released from the particles, or their synergistic activity.

**Table 1:** Types of metal-based nanoparticles, their applications, and toxicity.

MNPs	Usage	Toxicity
Aluminum oxide nanoparticles (AlO-NPs)	Cosmetics [26], Solid rocket propellants, Lubrication, and Drug delivery [27]	Neurotoxicity [28,29]
Cadmium oxide nanoparticles (CdO-NPs)	Paint pigments [30], Solar cells, and Phototransistors [31]	Genotoxicity [30], Developmental toxicity [32]
Copper nanoparticles (Cu-NPs)	Wastewater treatment [33]	Reproductive toxicity [34]
Copper oxide nanoparticles (CuO-NPs)	Nanofertilizers [35], Antifungal and antibacterial agent [36], Food packaging [37]	Respiratory toxicity [38], Neurotoxicity [39]
Gold nanoparticles (Au-NPs)	Photothermal therapy [40], Gene delivery and Targeted drug delivery [41], Biolabels [42]	Hepatotoxicity [43,44]
Iron oxide nanoparticles ^1^ (IO-NPs)	Cancer immunotherapy [45], Drug delivery [46]	Neurotoxicity [47,48]
Nickel oxide nanoparticles (NiO-NPs)	Lithium-ion batteries [49], Fuel cells, Drug delivery, and Antibiotics [50]	Hepatotoxicity [51], Respiratory toxicity [52]
Palladium nanoparticles (Pd-NPs)	Organic catalysis, Fuel cells, Biosensors and Electrocatalysis [53]	Immunotoxicity [54]
Rhenium nanoparticles (Re-NPs)	Tumor treatment therapy and Coatings [55]	-
Silver nanoparticles (Ag-NPs)	Anticancer therapy [56], Antiinflammatory drugs and Antibiotics [57]	Developmental toxicity [58], Genotoxicity [59], Hepatotoxicity [60]
Titanium dioxide nanoparticles (TiO_2_-NPs)	Photodynamic therapy [61], Toothpaste [62], Food additives [63], Sunscreen [64]	Respiratory toxicity [65], Neurotoxicity [66], Developmental toxicity [67]
Zinc oxide nanoparticles (ZnO-NPs)	Cosmetics, Sunscreen and Textile finishes [68], Drug carriers [69], Food packaging [26]	Developmental toxicity [70], Respiratory toxicity [71], Immunotoxicity [72]

^1^ IO-NPs represent Fe_2_O_3_ and Fe_3_O_4_ nanoparticles.

## 2. Exposure and Biodistribution of MNPs

Fine MNPs in the air are inhaled into the respiratory tract [73]. Welders who perform metal joining in the industrial manufacturing sector can easily inhale ultrafine metal particles, which can lead to several health problems. The inhalation of zinc oxide nanoparticles (ZnO-NPs) during welding contributes to metal fume fever, an occupational disease [74,75]. Due to their unique characteristics and benefits, various types of MNPs are used in the food industry. Among them, metal oxide nanoforms, including copper oxide nanoparticles, triiron tetraoxide nanoparticles, magnesium oxide nanoparticles, titanium dioxide nanoparticles (TiO_2_-NPs), and ZnO-NPs, are used in food nanopackaging due to their advantages over general metal nanoparticles [76,77,78]. Therefore, humans intake MNPs through food contaminated with nanoparticles. In the medical field, MNPs are utilized for biomedical applications, including diagnosis, assessment, and treatment, as well as the development of new diagnostic, therapeutic, and prognostic methods [79,80]. Gold nanoparticles (Au-NPs) were approved for use in genetic technology by the Food and Drug Administration [81]. Superparamagnetic iron oxide nanoparticles have been introduced as safe and efficient magnetic resonance imaging (MRI) contrast agents for therapeutic evaluation and targeted molecule targeting [82]. Despite disagreements regarding long-term safety and risks, the use of MNPs in cancer and hereditary diseases is growing. Accordingly, MNPs are present everywhere and enter the body via various routes.

### 2.1. TiO_2_-NPs

In the UK, the ingestion of TiO_2_-NPs was estimated at 37.5 mg (median), indicating approximately 0.04 mg/kg body weight per day for a 70 kg adult. Similar to the UK, people over 7 years of age intake 0.06–0.17 mg of TiO_2_-NPs/kg body weight per day [83]. When mice were orally injected with TiO_2_-NPs, accumulated TiO_2_-NPs were identified in the lymphoid tissue [84]. After oral ingestion of 6.25, 62.5, or 625 mg TiO_2_-NPs/kg body weight for 18 weeks, the particles were observed in the basal cells of murine Peyer’s patches. Additionally, TiO_2_-NPs can translocate into the circulatory system and move to various new targets, including brain microvessels [85]. In rats, an intravenous (IV) injection was administered to evaluate the biodistribution of TiO_2_-NPs. The liver, spleen, and lungs significantly accumulated TiO_2_-NPs after IV administration, showing significantly increased titanium concentrations in organs compared with non-injected rats from 30 min to 1 year. In addition, TiO_2_-NPs have been detected in the circulatory system after IV administration. In the brain microvasculature endothelial cells, the titanium burden was significantly detected from 5 min, which was approximately six-fold higher than that in the non-injected groups. Moreover, 24 h after TiO_2_-NPs injection, a significant accumulation of TiO_2_-NPs in the brain was observed. Mabondzo et al. suggested that titanium concentration in the injected group (261.40 ± 28.86 ng/g) was higher than that of the control group (68.25 ± 6.56 ng/g) 5 min after IV administration of TiO_2_-NPs [85]. While TiO_2_-NPs showed a significant toxic effect using rodents, there is a limit to completely extrapolating these results to humans. They are considered different conditions because there are differences in physiology, metabolism, genetics, biochemistry, diet, and environment between animals and humans. In addition, since animal experiments have limitations in reflecting the actual routes and amounts of exposure, the results of animal experiments are carefully interpreted.

### 2.2. Ag-NPs

Although the occupational exposure limit of Ag-NPs is 0.19 μg/m^3^, the concentration of Ag-NPs reached up to 1.35 μg/m^3^ during manufacturing and integration [86]. Since Ag-NPs have applications in various fields, including biomedical and commercial applications, they can enter the body via inhalation, ingestion, and contact [87]. Long-term exposure of Ag-NPs to medaka fish, from the embryo stage to adulthood, resulted in the accumulation of silver in various tissues, including the liver, gills, intestine, ovary, and brain [88]. In mice, the concentrations of silver in the lungs, heart, brain, kidneys, spleen, and liver were measured after one and seven days following intratracheal (IT) instillation [89]. Although the concentrations of silver in the heart, kidney, spleen, and liver decreased, the brain and lungs showed either the same or increased concentrations of silver on day 7 compared with those on day 1. Lee et al. demonstrated altered gene expression in the brains of mice administered 1.91 × 10^7^ particles/cm^3^ of 20 nm Ag-NPs via nose only [90].

### 2.3. Au-NPs

The blood and urine from the healthy male who inhaled 116 ± 12 μg/m^3^ of Au-NPs for 2 h contained a detectable concentration of gold [91]. Moreover, 24 h after inhalation, most subjects presented with gold in their bloodstream. In addition, approximately 1 ng/g (blood) and 100 ng/L (urine) of gold were detected for up to three months. Depending on their size, small Au-NPs were accumulated in the blood, urine, and liver when injected into mice through pulmonary instillation. Administration routes via injection, including IT instillation, gavage, and IV, are crucial for Au-NP accumulation in secondary target organs, such as the liver, spleen, kidney, brain, and others, as well as in the blood [92,93]. In cases of IT instillation and IV injection, none of the secondary target organs showed an increased Au-NP uptake at 24 h compared to 1 h after application. In contrast, gavage injection of Au-NPs resulted in longer detection in secondary target organs, excluding the liver, at 24 h compared to 1 h [92]. Since Au-NPs can enter the body through inhalation, ingestion, and direct injection into the bloodstream via nanomedicine applications, these data reflect the primary exposure routes of Au-NPs in humans. Nevertheless, since biological and interspecies differences exist between rodents and humans, animal experimental results have limitations in fully extrapolating to human toxicity.

### 2.4. ZnO-NPs

Inhalation of ZnO-NPs causes airway inflammation and metal fume fever [94]. Mice incubated in the chamber to inhale 1.93 × 10^6^/cm^3^ of ZnO-NPs for 3 days showed significantly increased Zn content [95]. Zn concentrations deposited in the group exposed to ZnO-NPs were measured at approximately 20.6 μg/g, which was 1.5-fold higher than that of the control group. Similar to these results, the Zn concentration was immediately increased in bronchoalveolar lavage fluid isolated from mice incubated in a whole-body chamber to inhale 3.6 ± 0.5 mg/m^3^ of ZnO-NPs [96]. The exposed and sham groups showed approximately 73 μg/L and 16 μg/L Zn, respectively. A significant (1.2-fold) increase in Zn concentration in the lungs of exposed mice compared with sham mice was observed. In addition to the respiratory tract, a higher Zn concentration was observed in the heart of mice exposed to ZnO-NP inhalation (approximately 160 μg/g vs. 115 μg/g). While single-gavage of ZnO-NPs did not significantly alter the Zn concentration in rats, multiple-gavage of ZnO-NPs for 90 days resulted in an increased Zn concentration in the bones compared with that in control rats [97].

### 2.5. Iron Oxide Nanoparticles (IO-NPs)

Iron oxide-based nanoparticles are mainly divided into maghemite (Fe_2_O_3_) and magnetite (Fe_3_O_4_) nanoparticles. In this review, IO-NPs represent both the Fe_2_O_3_ and Fe_3_O_4_ nanoparticles. IO-NPs are widely used in the medical field, including magnetic drug delivery, replacement therapy, and hyperthermia targeting specific organs [98]. Shan et al. showed that IV-injected IO-NPs mainly accumulated in the mouse liver (23.7% of injected dose (ID)/g) and spleen (12.6% ID/g) at an early point (0–6 h) based on the nanoparticle biodistribution coefficient [99]. This is consistent with biodistribution, hepatotoxicity, and pulmonary toxicity in rats after IO-NP inhalation [100]. IO-NPs were detected in the lungs, heart, kidneys, muscles, and brain, showing slower absorption and lower uptake in mice. Additionally, particle size plays a role in the accumulation and clearance of IO-NPs in the bloodstream [101]. The clearance of particles smaller than 100 nm from the bloodstream was more rapid than that of particles larger than 100 nm. However, IO-NPs from 100 to 200 nm in size circulated longer in the bloodstream and were retained in vascular fenestrations.

## 3. Cellular Effects of MNPs in the Cardiovascular System

### 3.1. Cell Death

#### 3.1.1. Apoptosis

Cellular death is broadly categorized into programmed cell death, which includes apoptosis, and non-programmed cell death [102]. Apoptosis is the process of programmed cell death to eliminate unwanted cells. Apoptosis requires energy and plays a crucial role in the pathogenesis of cardiovascular diseases [103,104]. Palladium nanoparticles induced apoptosis in human cardiac microvascular endothelial cells (HCMECs) in endothelial cell models [105]. However, TiO_2_-NP exposure caused severe apoptosis in HCMECs when treated to the same extent as palladium nanoparticles. In addition, human umbilical vein endothelial cells (HUVECs) underwent apoptosis induced by TiO_2_-NPs as determined by apoptotic markers, including Bax, Bcl-2, and the caspase family [106]. The apoptotic pathways are composed of the extrinsic pathway, initiated by signals originating from outside the cell, and the intrinsic pathway, which occurs within the injured cell [107]. Mitochondria are essential initiators of the intrinsic apoptosis pathway, activating intracellular caspase proteases that control cell death [108]. Human brain microvascular endothelial cells (HBMECs) and HUVECs were identified to exhibit mitochondrial damage induced by aluminum nanoparticles and TiO_2_-NPs, respectively [109,110]. Both studies suggested that nanoparticles have toxic effects on mitochondria, as evidenced by decreased mitochondrial membrane potential, altered mitochondrial morphology, and reduced intracellular ATP levels. Consistent with mitochondrial dysfunction, aluminum nanoparticles and TiO_2_-NPs significantly decreased cell viability as determined using the MTT assay for the detection of mitochondrial activity.

#### 3.1.2. Ferroptosis

Ferroptosis is a type of programmed cell death dependent on iron ion levels and increased intracellular lipid peroxidation [111]. Ferroptosis affects various cells of the cardiovascular system, including cardiac and vascular cells, and it has garnered attention as a novel risk factor and therapeutic target for cardiovascular diseases [112]. In HUVECs, IN-OPs and ZnO-NPs induced ferroptosis through upregulating phospholipid peroxides [113,114]. Along with cellular ferroptosis, ZnO-NPs promoted ferritinophagy and vascular inflammation mediated by ferroptosis in mouse models [114].

### 3.2. Oxidative Stress

Oxidative stress is caused by the imbalance between free radicals and antioxidants [115]. Several intracellular oxidative stress markers exist, including reactive oxygen species (ROS), serum superoxide dismutase, catalase, malondialdehyde, and glutathione [116,117]. Oxidative stress plays an essential role in the development and progression of cardiovascular diseases such as myocardial infarction, ischemia/reperfusion, and heart failure [118,119,120,121]. Brain endothelial (bEnd.3) cells, a component of the BBB, promoted oxidative stress induced by Ag-NPs as determined by measuring intracellular ROS levels [122]. In addition to bEnd.3 cells, small-sized Ag-NPs (5 nm) led to increased levels of heat shock protein 70 kDa (HSP-70) and heme oxygenase-1 protein, which is related to intracellular ROS levels in EA.hy926 cells (human umbilical vein cells) compared with 100 nm-sized particles [123]. Intensive intracellular oxidative stress contributes to cell death via necrotic, apoptotic, or autophagic pathways. The promotion of apoptosis was mediated by the increased intracellular oxidative stress in HUVECs exposed to IO-NPs, Ag-NPs, or TiO_2_-NPs [124,125,126].

### 3.3. Hyperpermeability

The endothelium acts as a physical barrier that separates and protects the surrounding tissues from the bloodstream [127]. The endothelial barrier function is facilitated by the selective vascular permeability, which is due to tight and adherent junction proteins [128]. Dysfunctional endothelium is the first step in vascular disease, leading to the injury of other cardiovascular cells [129]. Increased endothelial permeability is associated with the development and aggravation of cardiovascular diseases such as atherosclerosis, hypertension, stroke, and heart diseases. Brain endothelial cells are significant components of the BBB, acting as physical barriers and contributing to a highly selective, semipermeable membrane [130]. BBB plays a critical role in maintaining brain homeostasis and neurovascular health. The reduction in tight junction proteins and increased permeability result in a disrupted BBB, which contributes to neurovascular and cardiovascular damage. As brain endothelial cells prevent injury and toxicity to the central nervous system, BBB disruption leads to cardiovascular diseases and neurological dysfunction, including neurodegenerative diseases [131]. Aluminum nanoparticles and Ag-NPs reduced the expression of tight junction proteins, including ZO-1, occludin, and claudin-5, in HBMECs and bEnd.3, respectively [109,122]. Consistent with the decrease in tight junction protein expression, endothelial permeability increased in an in vitro BBB model exposed to Ag-NPs and TiO_2_-NPs [132,133].

### 3.4. Procoagulant Activity

Red blood cells (RBCs) are representative blood cells that transport oxygen from the lungs to body tissues [134]. Injured RBCs with altered functions have implications for cardiovascular damage and diseases, such as venous thrombosis, cardiac injury, and ischemia/reperfusion in diabetes [135,136,137]. Increased procoagulant activity of RBCs, characterized by the externalization of phosphatidylserine (PS) on the RBC membrane, is a key factor in triggering various pathological circulatory conditions, including thrombus formation, venous thrombosis, and erythrophagocytosis by immune and endothelial cells [136,138]. The significance of PS-exposed blood cells in cardiovascular diseases has also been demonstrated in clinical patients [139,140]. Exposure of human RBCs to Ag-NPs, ZnO-NPs, and TiO_2_-NPs increased the procoagulant and thrombotic activities associated with venous thrombosis [141,142,143]. ZnO-NP-exposed RBCs exhibited endothelial hyperpermeability and cytotoxicity attributed to excessive erythrophagocytosis by brain endothelial cells [141].

## 4. Pathological Conditions Induced by MNPs in the Cardiovascular System

### 4.1. Inflammation

Systemic and local inflammation plays an essential role in the development and progression of cardiovascular diseases [144]. IO-NP treatment promotes inflammation in human whole blood by increasing inflammatory cytokines, such as tumor necrosis factor (TNF)-α, interleukin (IL)-6, IL-1β, and IL-8 [145]. Along with whole blood, endothelial cells incubated with whole blood in combination with IO-NPs showed significantly increased levels of TNF-α, IL-6, IL-1β, and IL-8, indicating cardiovascular inflammation. Non-human primates and healthy persons exhibited systemic inflammation and metal fume fever after exposure to ZnO-NPs via IT injection or inhalation routes, respectively [146,147]. Inflammation plays a critical role in the pathophysiology of atherosclerosis and its transition into chronic inflammation [148]. Human aortic endothelial cells (HAECs) showed significantly increased nitric oxides and inflammatory factors, including intercellular adhesion molecule 1, IL-8, and monocyte chemoattractant protein-1 after treatment of IO-NPs, yttrium oxide nanoparticles, ZnO-NPs, and cerium oxide nanoparticles [149,150,151]. IO-NP-treated HAECs suggested that endothelial inflammation and dysfunction were promoted through increased cytokine expression, adhesion molecule expression, monocyte recruitment, and nitric oxide production, indicating risks for atherosclerosis [149].

### 4.2. Cardiotoxicity

Cardiovascular diseases encompass a range of cardiac and blood vessel disorders. The heart is the main secondary target organ of nanoparticles translocated from the circulatory system [8]. The oral uptake of MNPs, including IO-NPs, Ag-NPs, ZnO-NPs, and aluminum nanoparticles, has been shown to cause cardiotoxicity in animal models [26,152]. Long-term exposure to IO-NPs, Ag-NPs, or their mixture induced oxidative stress and increased the levels of pro-inflammatory cytokines such as TNF-α and IL-6 in cardiac tissues [152]. Additionally, myocardial degeneration occurred in isolated hearts from rats that led to changes in the myocardial fibers. IO-NPs promoted ROS levels in the cardiac tissues and had more fatal effects on mouse survival after a single IV injection than that of Au-NPs [153]. The heart weight is crucial in several age-related pathological conditions and cardiovascular diseases. After long-term exposure to ZnO-NPs, aluminum nanoparticles, or their combination, the heart weight decreased, and levels of TNF-α and IL-6 increased in cardiac tissues isolated from rats [26].

### 4.3. Vascular Toxicity

The circulatory system consists of vessels that carry blood and lymphatic fluid. The vascular system is continuously in contact with the absorbed NPs during the translocation of NPs from primary to secondary target organs. Additionally, MNPs, such as ZnO-NPs and TiO_2_-NPs, are attached to and located within circulating RBCs [154]. In rats, IV injections of nickel nanoparticles into the dorsal penile vein resulted in an altered lipid profile, as indicated by increased cholesterol and apolipoprotein E levels, which are associated with atherosclerosis [155]. Blood cells in the bloodstream contribute to vasculopathy, such as venous thrombosis. IV injection of Ag-NPs, TiO_2_-NPs, or ZnO-NPs led to thrombus formation in the veins in an in vivo rat venous model [141,142,143]. When rats were exposed to TiO2-NPs for 30 days via the oral route, the counts of white blood cells and granulocytes in the blood increased [156]. Moreover, TiO_2_-NP exposure in rats initiated an inflammatory response, as shown by the increased concentrations of TNF-α and IL-6 in the serum.

## 5. Comparative Toxicity Between Metal Ions and MNPs

Because MNPs are derived from metals, they have unique characteristics due to the release of nano-sized metal ions. In experimental models, MNPs are dissolved in aqueous media, releasing metal ions into the surrounding media [157]. To identify the potential risks of MNPs, it is necessary to understand whether the toxic effects are caused by the particles themselves, the released metal ions, or their combination. Different perspectives on MNP-induced toxicity exist; some studies suggest that released ions are the primary or sole cause of toxic effects [158,159], whereas other studies suggest that particles, rather than released ions from MNPs, are the major contributors to toxic effects [160] (Table 2).

Metal salts, such as ZnCl_2_, are distributed into various tissues through circulation rather than ZnO-NPs [161]. In addition, while oral uptake of CuCl_2_ induced toxicity within 24 h, it was observed that the toxic effects induced by copper nanoparticles were delayed until 48 h [162]. Rats orally injected with Ag-NPs showed no pathological symptoms. However, silver acetate (AgOAc) injection led to toxic effects, including weight loss, linitis plastica, tissue pigmentation, and death in animal models [163]. ZnCl_2_, which has a lower zinc concentration than ZnO-NPs, caused severe toxicity and led to pathological conditions, such as anemia and tissue injuries in the liver, kidneys, lungs, and intestine [161].

**Figure 1:**
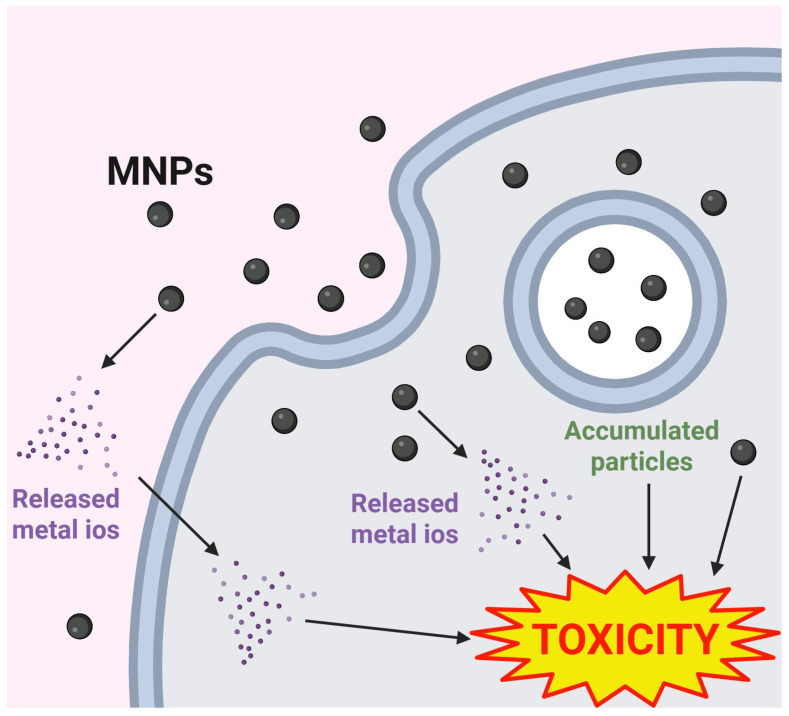
Comparison of MNP pathways contributing to toxicity. MNPs lead to toxic effects through metal ions, particles per se, or their combination. MNPs release their metal ions in aqueous media or the cytoplasm. Entered particles are accumulated in the organelle, as indicated by the released ions. These released ions and intracellular accumulated particles participate in the toxicity induced by MNPs.

Although metal ions released from MNPs primarily contribute to acute toxicity in animal models, particles accumulate in tissues and cells for more extended periods than metal salts [122,162]. ZnO-NPs and TiO2-NPs were identified as the primary sources that induced hemolysis of RBCs and oxidative stress rather than their released ions [164]. In addition, the cytotoxicity of ZnO-NPs resulted from the particles rather than the released zinc ions in glial cells [165]. Lower concentrations of ZnO-NPs induced changes in cellular morphology and showed increased cell death compared with ZnSO_4_. The increased concentrations of intracellular zinc ions and silver ions derived from accumulated particles were identified in ZnO-NPs-treated HUVECs and Ag-NPs-treated bEnd.3 cells compared with treatments with ZnCl_2_ and Ag ions alone, respectively [122,166]. In a study on the procoagulant activity of ZnO-NPs on RBCs, TPEN, a zinc chelator, promoted PS exposure to a certain extent by binding to zinc ions released from ZnO-NPs [141]. However, this study had a limitation in that the concentration of Zn ions released from ZnCl_2_ treatment differed from that released from ZnO-NPs due to the pH of the surrounding media. These reports suggest that the accumulation of particles in cells and tissues, as well as the concentration of released ions, are closely related, and MNP-induced toxicity can be attributed to either factor or their synergistic activation (Figure 1).

## 6. Future Directions

**Figure 2:**
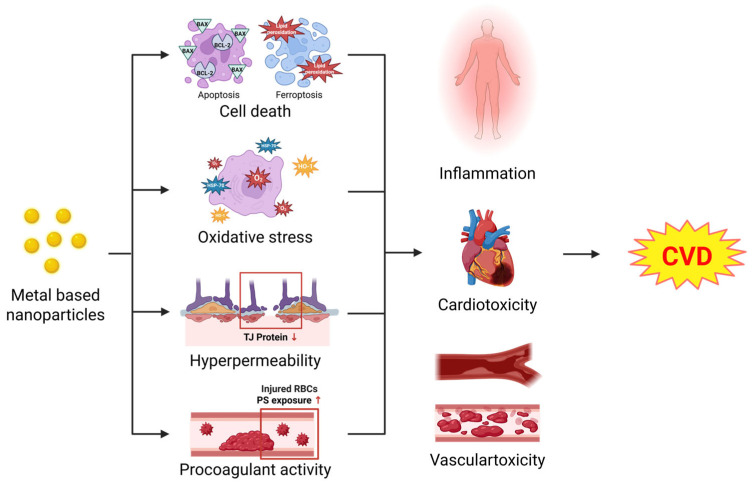
Cardiovascular diseases caused by MNPs. Circulating MNPs induce cellular death, oxidative stress, endothelial dysfunction, hyperpermeability, as well as procoagulant activity in the cardiovascular system. Cytotoxicity and altered cellular functions are closely associated with inflammation, cardiotoxicity, and vascular toxicity. These pathological conditions result from MNPs contributing to the development of cardiovascular diseases.

These studies support that MNPs contribute to the development of cardiovascular diseases (Figure 2). Evidence indicates that the cardiovascular toxicity of MNPs is essential to establish an experimental model to explain this potential toxicity. Future studies should delineate the distinct toxic mechanisms of nanoparticles per se, released metal ions, and their combinations through carefully designed comparative studies. To provide different perspectives on cumulative risks, it is necessary to investigate the long-term biodistribution and persistence of MNPs in cardiovascular tissues.

To align nanotoxicology research more closely with regulatory sciences and enhance its utility for both academic and policy purposes, we suggest the following points. The incorporation of advanced methods for nanotoxicity assessment, including high-throughput screening (HTS), omics technologies (e.g., transcriptomics, proteomics, and metabolomics), and 3D organ-on-a-chip models, would offer valuable insights into the mechanistic understanding of nanoparticle-induced toxicity and facilitate predictive modeling. Additionally, the influence of surface modifications (e.g., PEGylation, ligand conjugation, or charge alteration) on the biological interactions and toxicity profiles of metal-based nanoparticles should be discussed. Surface chemistry plays a pivotal role in nanoparticle uptake, biodistribution, and immune response, thereby having direct implications for both safety and therapeutic potential.

Standard in vivo models and exposure conditions are required to simulate real-world human exposure more accurately, particularly in chronic low-dose exposure scenarios. To simulate the proper experimental models, we need to establish risk assessments of MNPs to acquire clear exposure quantifications. In addition, most current studies have several limitations in applying the actual exposure routes of MNPs. Therefore, it is essential to consider both the exposure routes and the exposure amount to implement the actual exposure scenario accurately. In addition, including real-world environmental and occupational exposure scenarios, such as inhalation risks in industrial settings or chronic low-dose exposure through consumer products, would provide a more grounded understanding of public health implications. There are currently regulatory measures and attempts being made specifically to consider nanoparticles worldwide. Despite these efforts, challenges persist in consistently defining nanoparticles, assessing real-world exposure, and establishing legally binding limits. As such, regulatory bodies worldwide continue to refine risk assessment methodologies, develop nano-specific safety guidelines, and promote safe-by-design principles to ensure the responsible development and use of metal-based nanomaterials. Through these further studies, we aim to highlight the “safe-by-design” strategies for nanoparticles that integrate safety considerations early in the material design phase, thereby minimizing adverse outcomes such as cardiovascular risks, which have been increasingly associated with nanoparticle exposure.

## Figures and Tables

**Table 2:** Representative examples of different biological effects between metal ions and particles per se.

Category	Metal-Ions	Particles
Distribution	Rapid distribution into various tissues via circulation	Accumulate in tissues and cells over extended periods
Toxicity onset	Rapid onset (e.g., CuCl_2_ toxicity within 24 h)	Delayed toxicity (e.g., Cu-NPs effects after 48 h)
Toxic mechanisms	Mainly due to free metal ions	Primarily caused by the particles per se (e.g., glial cells)
Pathological effects	Strong acute toxicity (e.g., AgOAc)	Some show no pathological symptoms (e.g., Ag-NPs)

## Abbreviations

AgOAc	Silver Acetate
Ag-NP	Silver Nanoparticle
Au-NP	Gold Nanoparticle
BBB	Blood–brain Barrier
HAEC	Human Aortic Endothelial Cell
HBMEC	Human Brain Microvascular Endothelial Cell
HCMEC	Human Cardiac Microvascular Endothelial Cell
HUVEC	Human Umbilical Vein Endothelial Cell
HSP	Heat Shock Protein
IL	Interleukin
IO-NP	Iron Oxide Nanoparticle
IT	Intratracheal
IV	Intravenous
MNP	Metal-based Nanoparticle
MRI	Magnetic Resonance Imaging
PS	Phosphatidylserine
RBC	Red Blood Cell
TiO_2_-NP	Titanium Dioxide Nanoparticle
TNF	Tumor Necrosis Factor
ZnO-NP	Zinc Oxide Nanoparticle

## References

[1] Malik S., Muhammad K., Waheed Y. (2023). Emerging Applications of Nanotechnology in Healthcare and Medicine. Molecules.

[2] Malik S., Muhammad K., Waheed Y. (2023). Nanotechnology: A Revolution in Modern Industry. Molecules.

[3] Saeed S., Ud Din S.R., Khan S.U., Gul R., Kiani F.A., Wahab A., Zhong M. (2023). Nanoparticle: A Promising Player in Nanomedicine and its Theranostic Applications for the Treatment of Cardiovascular Diseases. Curr. Probl. Cardiol..

[4] Gupta V., Mohapatra S., Mishra H., Farooq U., Kumar K., Ansari M.J., Aldawsari M.F., Alalaiwe A.S., Mirza M.A., Iqbal Z. (2022). Nanotechnology in Cosmetics and Cosmeceuticals-A Review of Latest Advancements. Gels.

[5] Ameen F., Alsamhary K., Alabdullatif J.A., Alnadhari S. (2021). A review on metal-based nanoparticles and their toxicity to beneficial soil bacteria and fungi. Ecotoxicol. Environ. Saf..

[6] Xuan L., Ju Z., Skonieczna M., Zhou P.K., Huang R. (2023). Nanoparticles-induced potential toxicity on human health: Applications, toxicity mechanisms, and evaluation models. MedComm.

[7] Yee M.S.-L., Hii L.-W., Looi C.K., Lim W.-M., Wong S.-F., Kok Y.-Y., Tan B.-K., Wong C.-Y., Leong C.-O. (2021). Impact of Microplastics and Nanoplastics on Human Health. Nanomaterials.

[8] Oberdorster G., Oberdorster E., Oberdorster J. (2005). Nanotoxicology: An emerging discipline evolving from studies of ultrafine particles. Environ. Health Perspect.

[9] Maher B.A., Ahmed I.A., Karloukovski V., MacLaren D.A., Foulds P.G., Allsop D., Mann D.M., Torres-Jardon R., Calderon-Garciduenas L. (2016). Magnetite pollution nanoparticles in the human brain. Proc. Natl. Acad. Sci. USA.

[10] Khan S.U., Suleman M., Khan M.U., Khan M.S., Arbi F.M., Hussain T., Alsuhaibani A.M., Refat M.S. (2024). Natural Allies for Heart Health: Nrf2 Activation and Cardiovascular Disease Management. Curr. Probl. Cardiol..

[11] Ullah M., Bibi A., Wahab A., Hamayun S., Rehman M.U., Khan S.U., Awan U.A., Riaz N.-U., Naeem M., Saeed S. (2024). Shaping the Future of Cardiovascular Disease by 3D Printing Applications in Stent Technology and its Clinical Outcomes. Curr. Probl. Cardiol..

[12] Li H., Chen X., Shen D., Wu F., Pleixats R., Pan J. (2021). Functionalized silica nanoparticles: Classification, synthetic approaches and recent advances in adsorption applications. Nanoscale.

[13] Burlec A.F., Corciova A., Boev M., Batir-Marin D., Mircea C., Cioanca O., Danila G., Danila M., Bucur A.F., Hancianu M. (2023). Current Overview of Metal Nanoparticles’ Synthesis, Characterization, and Biomedical Applications, with a Focus on Silver and Gold Nanoparticles. Pharmaceuticals.

[14] Zabeo A., Basei G., Tsiliki G., Peijnenburg W., Hristozov D. (2022). Ordered weighted average based grouping of nanomaterials with Arsinh and dose response similarity models. NanoImpact.

[15] Hardy A., Benford D., Halldorsson T., Jeger M.J., Knutsen H.K., More S., Naegeli H., Noteborn H., Ockleford C., Ricci A. (2018). Guidance on risk assessment of the application of nanoscience and nanotechnologies in the food and feed chain: Part 1, human and animal health. EFSA J.

[16] Petersen E.J., Ceger P., Allen D.G., Coyle J., Derk R., Garcia-Reyero N., Gordon J., Kleinstreuer N.C., Matheson J., McShan D. (2022). U.S. Federal Agency interests and key considerations for new approach methodologies for nanomaterials. ALTEX.

[17] Erdely A., Dahm M.M., Schubauer-Berigan M.K., Chen B.T., Antonini J.M., Hoover M.D. (2016). Bridging the gap between exposure assessment and inhalation toxicology: Some insights from the carbon nanotube experience. J. Aerosol. Sci..

[18] Yadavalli T., Shukla D. (2017). Role of metal and metal oxide nanoparticles as diagnostic and therapeutic tools for highly prevalent viral infections. Nanomedicine.

[19] Kornberg T.G., Stueckle T.A., Antonini J.A., Rojanasakul Y., Castranova V., Yang Y., Wang L. (2017). Potential Toxicity and Underlying Mechanisms Associated with Pulmonary Exposure to Iron Oxide Nanoparticles: Conflicting Literature and Unclear Risk. Nanomaterials.

[20] Morimoto Y., Izumi H., Tomonaga T., Nishida C., Higashi H. (2025). Adverse effects of nanoparticles on humans. J. Occup Health.

[21] Zhou X., Jin W., Ma J. (2023). Lung inflammation perturbation by engineered nanoparticles. Front. Bioeng Biotechnol..

[22] Wang Y.-L., Lee Y.-H., Chou C.-L., Chang Y.-S., Liu W.-C., Chiu H.-W. (2024). Oxidative stress and potential effects of metal nanoparticles: A review of biocompatibility and toxicity concerns. Environ. Pollut..

[23] Zhang N., Xiong G., Liu Z. (2022). Toxicity of metal-based nanoparticles: Challenges in the nano era. Front. Bioeng Biotechnol..

[24] Mortezaee K., Najafi M., Samadian H., Barabadi H., Azarnezhad A., Ahmadi A. (2019). Redox interactions and genotoxicity of metal-based nanoparticles: A comprehensive review. Chem. Biol. Interact.

[25] Xiong P., Huang X., Ye N., Lu Q., Zhang G., Peng S., Wang H., Liu Y. (2022). Cytotoxicity of Metal-Based Nanoparticles: From Mechanisms and Methods of Evaluation to Pathological Manifestations. Adv. Sci..

[26] Yousef M., Roychoudhury S., Jafaar K., Slama P., Kesari K., Kamel M. (2022). Aluminum oxide and zinc oxide induced nanotoxicity in rat brain, heart, and lung. Physiol. Res..

[27] Park S.-H., Lim J.-O., Kim W.-I., Park S.-W., Lee S.-J., Shin I.-S., Moon C., Kim J.-H., Heo J.-D., Kim J.-C. (2022). Subchronic Toxicity Evaluation of Aluminum Oxide Nanoparticles in Rats Following 28-Day Repeated Oral Administration. Biol. Trace Element Res..

[28] Huang T., Guo W., Wang Y., Chang L., Shang N., Chen J., Fan R., Zhang L., Gao X., Niu Q. (2021). Involvement of Mitophagy in Aluminum Oxide Nanoparticle-Induced Impairment of Learning and Memory in Mice. Neurotox Res..

[29] Zhang Y., Jia L., Wang Z., Guo W., Qin X., Ge C., Niu Q., Zhang Q. (2025). Alumina nanoparticles induce learning and memory impairment in a particle size-dependent and time-dependent manner. Ecotoxicol. Environ. Saf..

[30] Demir E., Qin T., Li Y., Zhang Y., Guo X., Ingle T., Yan J., Orza A.I., Biris A.S., Ghorai S. (2020). Cytotoxicity and genotoxicity of cadmium oxide nanoparticles evaluated using in vitro assays. Mutat. Res. Toxicol. Environ. Mutagen..

[31] Dabour K., Al Naggar Y., Masry S., Naiem E., Giesy J.P. (2019). Cellular alterations in midgut cells of honey bee workers (*Apis millefera* L.) exposed to sublethal concentrations of CdO or PbO nanoparticles or their binary mixture. Sci. Total. Environ..

[32] Blum J.L., Xiong J.Q., Hoffman C., Zelikoff J.T. (2012). Cadmium associated with inhaled cadmium oxide nanoparticles impacts fetal and neonatal development and growth. Toxicol. Sci..

[33] Ameh T., Sayes C.M. (2019). The potential exposure and hazards of copper nanoparticles: A review. Environ. Toxicol. Pharmacol..

[34] Anima B., Mondal P., Gurusubramanian G., Roy V.K. (2023). Mechanistic study of copper nanoparticle (CuNP) toxicity on the mouse uterus via apelin signaling. Environ. Sci. Pollut Res. Int..

[35] Devaraji M., Thanikachalam P.V., Elumalai K. (2024). The potential of copper oxide nanoparticles in nanomedicine: A comprehensive review. Biotechnol. Notes.

[36] Letchumanan D., Sok S.P.M., Ibrahim S., Nagoor N.H., Arshad N.M. (2021). Plant-Based Biosynthesis of Copper/Copper Oxide Nanoparticles: An Update on Their Applications in Biomedicine, Mechanisms, and Toxicity. Biomolecules.

[37] Dash K.K., Deka P., Bangar S.P., Chaudhary V., Trif M., Rusu A. (2022). Applications of Inorganic Nanoparticles in Food Packaging: A Comprehensive Review. Polymers.

[38] Zhang Y., Zhang Z., Mo Y., Zhang Y., Yuan J., Zhang Q. (2024). MMP-3 mediates copper oxide nanoparticle-induced pulmonary inflammation and fibrosis. J. Nanobiotechnol..

[39] Goma A.A., El Okle O.S., Tohamy H.G. (2021). Protective effect of methylene blue against copper oxide nanoparticle-induced neurobehavioral toxicity. Behav. Brain. Res..

[40] Singh P., Pandit S., Mokkapati V., Garg A., Ravikumar V., Mijakovic I. (2018). Gold Nanoparticles in Diagnostics and Therapeutics for Human Cancer. Int. J. Mol. Sci..

[41] Golchin K., Golchin J., Ghaderi S., Alidadiani N., Eslamkhah S., Eslamkhah M., Davaran S., Akbarzadeh A. (2018). Gold nanoparticles applications: From artificial enzyme till drug delivery. Artif. Cells Nanomed. Biotechnol..

[42] Daraee H., Eatemadi A., Abbasi E., Fekri Aval S., Kouhi M., Akbarzadeh A. (2016). Application of gold nanoparticles in biomedical and drug delivery. Artif Cells Nanomed. Biotechnol..

[43] Enea M., Pereira E., Costa J., Soares M.E., Dias da Silva D., Bastos M.L., Carmo H.F. (2021). Cellular uptake and toxicity of gold nanoparticles on two distinct hepatic cell models. Toxicol In Vitro.

[44] Yang Y., Fan S., Chen Q., Lu Y., Zhu Y., Chen X., Xia L., Huang Q., Zheng J., Liu X. (2023). Correction: Acute exposure to gold nanoparticles aggravates lipopolysaccharide-induced liver injury by amplifying apoptosis via ROS-mediated macrophage-hepatocyte crosstalk. J. Nanobiotechnol..

[45] Meng Y.Q., Shi Y.N., Zhu Y.P., Liu Y.Q., Gu L.W., Liu D.D., Ma A., Xia F., Guo Q.Y., Xu C.C. (2024). Recent trends in preparation and biomedical applications of iron oxide nanoparticles. J. Nanobiotechnol..

[46] Chamorro S., Gutierrez L., Vaquero M.P., Verdoy D., Salas G., Luengo Y., Brenes A., Jose Teran F. (2015). Safety assessment of chronic oral exposure to iron oxide nanoparticles. Nanotechnology.

[47] Dora M.F., Taha N.M., Lebda M.A., Hashem A.E., Elfeky M.S., El-Sayed Y.S., Jaouni S.A., El-Far A.H. (2021). Quercetin Attenuates Brain Oxidative Alterations Induced by Iron Oxide Nanoparticles in Rats. Int. J. Mol. Sci..

[48] Patel S., Jana S., Chetty R., Thakore S., Singh M., Devkar R. (2019). Toxicity evaluation of magnetic iron oxide nanoparticles reveals neuronal loss in chicken embryo. Drug Chem. Toxicol..

[49] More S.L., Kovochich M., Lyons-Darden T., Taylor M., Schulte A.M., Madl A.K. (2021). Review and Evaluation of the Potential Health Effects of Oxidic Nickel Nanoparticles. Nanomaterials.

[50] Berhe M.G., Gebreslassie Y.T. (2023). Biomedical Applications of Biosynthesized Nickel Oxide Nanoparticles. Int. J. Nanomed..

[51] Adiguzel C., Karaboduk H., Apaydin F.G., Kalender S., Kalender Y. (2023). Comparison of nickel oxide nano and microparticles toxicity in rat liver: Molecular, biochemical, and histopathological study. Toxicol. Res..

[52] Morimoto Y., Hirohashi M., Ogami A., Oyabu T., Myojo T., Hashiba M., Mizuguchi Y., Kambara T., Lee B.W., Kuroda E. (2011). Pulmonary toxicity following an intratracheal instillation of nickel oxide nanoparticle agglomerates. J. Occup. Health.

[53] Leso V., Iavicoli I. (2018). Palladium Nanoparticles: Toxicological Effects and Potential Implications for Occupational Risk Assessment. Int. J. Mol. Sci..

[54] Gurunathan S., Kang M.H., Jeyaraj M., Kim J.H. (2021). Palladium Nanoparticle-Induced Oxidative Stress, Endoplasmic Reticulum Stress, Apoptosis, and Immunomodulation Enhance the Biogenesis and Release of Exosome in Human Leukemia Monocytic Cells (THP-1). Int. J. Nanomed..

[55] Kepceoglu A., Gundogdu Y., Sarilmaz A., Ersoz M., Ozel F., Kilic H.S. (2021). Rhenium/rhenium oxide nanoparticles production using femtosecond pulsed laser ablation in liquid. Turk. J. Chem..

[56] Xu L., Wang Y.Y., Huang J., Chen C.Y., Wang Z.X., Xie H. (2020). Silver nanoparticles: Synthesis, medical applications and biosafety. Theranostics.

[57] Almatroudi A. (2024). Unlocking the Potential of Silver Nanoparticles: From Synthesis to Versatile Bio-Applications. Pharmaceutics.

[58] Quevedo A.C., Lynch I., Valsami-Jones E. (2021). Silver nanoparticle induced toxicity and cell death mechanisms in embryonic zebrafish cells. Nanoscale.

[59] AshaRani P.V., Low Kah Mun G., Hande M.P., Valiyaveettil S. (2009). Cytotoxicity and genotoxicity of silver nanoparticles in human cells. ACS Nano.

[60] Assar D.H., Mokhbatly A.-A.A., Ghazy E.W., Elbialy Z.I., Gaber A.A., Hassan A.A., Nabil A., Asa S.A. (2022). Silver nanoparticles induced hepatoxicity via the apoptotic/antiapoptotic pathway with activation of TGFbeta-1 and alpha-SMA triggered liver fibrosis in Sprague Dawley rats. Environ. Sci. Pollut Res. Int..

[61] Cesmeli S., Biray Avci C. (2019). Application of titanium dioxide (TiO_2_) nanoparticles in cancer therapies. J. Drug Target..

[62] Shakeel M., Jabeen F., Shabbir S., Asghar M.S., Khan M.S., Chaudhry A.S. (2016). Toxicity of Nano-Titanium Dioxide (TiO_2_-NP) Through Various Routes of Exposure: A Review. Biol. Trace Element Res..

[63] Baranowska-Wojcik E., Szwajgier D., Oleszczuk P., Winiarska-Mieczan A. (2020). Effects of Titanium Dioxide Nanoparticles Exposure on Human Health-a Review. Biol. Trace Element Res..

[64] Schneider S.L., Lim H.W. (2019). A review of inorganic UV filters zinc oxide and titanium dioxide. Photodermatol. Photoimmunol. Photomed..

[65] Jeong J.S., Jegal H., Ko J.W., Kim J.W., Kim J.H., Chung E.H., Boo S.Y., Lee S.H., Lee G.W., Park S.M. (2025). Titanium Dioxide Nanoparticle Exposure Provokes Greater Lung Inflammation in Females Than Males in the Context of Obesity. Int. J. Nanomed..

[66] Eid A., Ghaleb S.S., Zaki A., Ibrahim M., Farghali A.A., Ali L.E., Abdelgawad M.A., Ghoneim M.M., Al-Serwi R.H., Hassan R.M. (2023). Hesperidin Attenuates Titanium Dioxide Nanoparticle-Induced Neurotoxicity in Rats by Regulating Nrf-2/TNF-alpha Signaling Pathway, the Suppression of Oxidative Stress, and Inflammation. ACS Omega.

[67] Mohamadzadeh N., Zirak Javanmard M., Karimipour M., Farjah G. (2021). Developmental Toxicity of the Neural Tube Induced by Titanium Dioxide Nanoparticles in Mouse Embryos. Avicenna J. Med. Biotechnol..

[68] Jiang J., Pi J., Cai J. (2018). The Advancing of Zinc Oxide Nanoparticles for Biomedical Applications. Bioinorg. Chem. Appl..

[69] Mishra P.K., Mishra H., Ekielski A., Talegaonkar S., Vaidya B. (2017). Zinc oxide nanoparticles: A promising nanomaterial for biomedical applications. Drug Discov. Today.

[70] Kteeba S.M., El-Adawi H.I., El-Rayis O.A., El-Ghobashy A.E., Schuld J.L., Svoboda K.R., Guo L. (2017). Zinc oxide nanoparticle toxicity in embryonic zebrafish: Mitigation with different natural organic matter. Environ. Pollut.

[71] Morimoto Y., Izumi H., Yoshiura Y., Tomonaga T., Oyabu T., Myojo T., Kawai K., Yatera K., Shimada M., Kubo M. (2016). Evaluation of Pulmonary Toxicity of Zinc Oxide Nanoparticles Following Inhalation and Intratracheal Instillation. Int. J. Mol. Sci..

[72] Senapati V.A., Gupta G.S., Pandey A.K., Shanker R., Dhawan A., Kumar A. (2017). Zinc oxide nanoparticle induced age dependent immunotoxicity in BALB/c mice. Toxicol. Res..

[73] Andujar P., Lanone S., Brochard P., Boczkowski J. (2011). Respiratory effects of manufactured nanoparticles. Rev. Mal. Respir..

[74] Szucs-Somlyo E., Lehel J., Majlinger K., Lorincz M., Kovago C. (2023). Metal-oxide inhalation induced fever-Immuntoxicological aspects of welding fumes. Food Chem. Toxicol..

[75] Monse C., Raulf M., Jettkant B., van Kampen V., Kendzia B., Schurmeyer L., Seifert C.E., Marek E.M., Westphal G., Rosenkranz N. (2021). Health effects after inhalation of micro- and nano-sized zinc oxide particles in human volunteers. Arch. Toxicol..

[76] Adeyemi J.O., Fawole O.A. (2023). Metal-Based Nanoparticles in Food Packaging and Coating Technologies: A Review. Biomolecules.

[77] Zhou X., Zhou X., Zhou L., Jia M., Xiong Y. (2024). Nanofillers in Novel Food Packaging Systems and Their Toxicity Issues. Foods.

[78] Hashemifard Dehkordi P., Moshtaghi H., Abbasvali M. (2023). Effects of magnesium oxide and copper oxide nanoparticles on biofilm formation ofEscherichia coliandListeria monocytogenes. Nanotechnology.

[79] Roshani M., Rezaian-Isfahni A., Lotfalizadeh M.H., Khassafi N., Abadi M., Nejati M. (2023). Metal nanoparticles as a potential technique for the diagnosis and treatment of gastrointestinal cancer: A comprehensive review. Cancer Cell Int..

[80] Scafa Udriste A., Burdusel A.C., Niculescu A.G., Radulescu M., Grumezescu A.M. (2024). Metal-Based Nanoparticles for Cardiovascular Diseases. Int. J. Mol. Sci..

[81] Sibuyi N.R.S., Moabelo K.L., Fadaka A.O., Meyer S., Onani M.O., Madiehe A.M., Meyer M. (2021). Multifunctional Gold Nanoparticles for Improved Diagnostic and Therapeutic Applications: A Review. Nanoscale Res. Lett..

[82] Wahajuddin, Arora S. (2012). Superparamagnetic iron oxide nanoparticles: Magnetic nanoplatforms as drug carriers. Int. J. Nanomed..

[83] Lomer M.C., Hutchinson C., Volkert S., Greenfield S.M., Catterall A., Thompson R.P., Powell J.J. (2004). Dietary sources of inorganic microparticles and their intake in healthy subjects and patients with Crohn’s disease. Br. J. Nutr..

[84] Riedle S., Wills J.W., Miniter M., Otter D.E., Singh H., Brown A.P., Micklethwaite S., Rees P., Jugdaohsingh R., Roy N.C. (2020). A Murine Oral-Exposure Model for Nano- and Micro-Particulates: Demonstrating Human Relevance with Food-Grade Titanium Dioxide. Small.

[85] Disdier C., Devoy J., Cosnefroy A., Chalansonnet M., Herlin-Boime N., Brun E., Lund A., Mabondzo A. (2015). Tissue biodistribution of intravenously administrated titanium dioxide nanoparticles revealed blood-brain barrier clearance and brain inflammation in rat. Part. Fibre Toxicol..

[86] Nie P., Zhao Y., Xu H. (2023). Synthesis, applications, toxicity and toxicity mechanisms of silver nanoparticles: A review. Ecotoxicol. Environ. Saf..

[87] Ferdous Z., Nemmar A. (2020). Health Impact of Silver Nanoparticles: A Review of the Biodistribution and Toxicity Following Various Routes of Exposure. Int. J. Mol. Sci..

[88] Yan N., Wang W.X. (2022). Maternal transfer and biodistribution of citrate and luminogens coated silver nanoparticles in medaka fish. J. Hazard. Mater..

[89] Ferdous Z., Al-Salam S., Yuvaraju P., Ali B.H., Nemmar A. (2021). Remote effects and biodistribution of pulmonary instilled silver nanoparticles in mice. NanoImpact.

[90] Lee H.-Y., Choi Y.-J., Jung E.-J., Yin H.-Q., Kwon J.-T., Kim J.-E., Im H.-T., Cho M.-H., Kim J.-H., Kim H.-Y. (2010). Genomics-based screening of differentially expressed genes in the brains of mice exposed to silver nanoparticles via inhalation. J. Nanopart. Res..

[91] Miller M.R., Raftis J.B., Langrish J.P., McLean S.G., Samutrtai P., Connell S.P., Wilson S., Vesey A.T., Fokkens P.H., Boere A.J.F. (2017). Inhaled Nanoparticles Accumulate at Sites of Vascular Disease. ACS Nano.

[92] Schmid G., Kreyling W.G., Simon U. (2017). Toxic effects and biodistribution of ultrasmall gold nanoparticles. Arch. Toxicol..

[93] Irvin-Choy N.S., Nelson K.M., Dang M.N., Gleghorn J.P., Day E.S. (2021). Gold nanoparticle biodistribution in pregnant mice following intravenous administration varies with gestational age. Nanomedicine.

[94] Huang K.L., Chang H.L., Tsai F.M., Lee Y.H., Wang C.H., Cheng T.J. (2019). The effect of the inhalation of and topical exposure to zinc oxide nanoparticles on airway inflammation in mice. Toxicol. Appl. Pharmacol..

[95] Rossner P., Vrbova K., Strapacova S., Rossnerova A., Ambroz A., Brzicova T., Libalova H., Javorkova E., Kulich P., Vecera Z. (2019). Inhalation of ZnO Nanoparticles: Splice Junction Expression and Alternative Splicing in Mice. Toxicol. Sci..

[96] Adamcakova-Dodd A., Stebounova L.V., Kim J.S., Vorrink S.U., Ault A.P., O’sHaughnessy P.T., Grassian V.H., Thorne P.S. (2014). Toxicity assessment of zinc oxide nanoparticles using sub-acute and sub-chronic murine inhalation models. Part. Fibre Toxicol..

[97] Liang C., Fang J., Hu J., Geng X., Liu H., Feng Y., Wang W., Cui W., Yu Z., Jia X. (2022). Toxicokinetics of zinc oxide nanoparticles and food grade bulk-sized zinc oxide in rats after oral dosages. NanoImpact.

[98] Dadfar S.M., Roemhild K., Drude N.I., von Stillfried S., Knuchel R., Kiessling F., Lammers T. (2019). Iron oxide nanoparticles: Diagnostic, therapeutic and theranostic applications. Adv. Drug Deliv. Rev..

[99] Kumar M., Kulkarni P., Liu S., Chemuturi N., Shah D.K. (2023). Nanoparticle biodistribution coefficients: A quantitative approach for understanding the tissue distribution of nanoparticles. Adv. Drug Deliv. Rev..

[100] Sadeghi L., Yousefi Babadi V., Espanani H.R. (2015). Toxic effects of the Fe_2_O_3_ nanoparticles on the liver and lung tissue. Bratisl. Lek. Listy.

[101] Alphandery E. (2019). Biodistribution and targeting properties of iron oxide nanoparticles for treatments of cancer and iron anemia disease. Nanotoxicology.

[102] Shen S., Shao Y., Li C. (2023). Different types of cell death and their shift in shaping disease. Cell Death Discov..

[103] Elmore S. (2007). Apoptosis: A review of programmed cell death. Toxicol. Pathol..

[104] Lee Y., Gustafsson A.B. (2009). Role of apoptosis in cardiovascular disease. Apoptosis.

[105] Fromell K., Johansson U., Abadgar S., Bourzeix P., Lundholm L., Elihn K. (2023). The effect of airborne Palladium nanoparticles on human lung cells, endothelium and blood-A combinatory approach using three in vitro models. Toxicol. Vitr..

[106] Zeng C., Feng Y., Wang W., Zhou F., Liao F., Liu Y., Feng S. (2018). The size-dependent apoptotic effect of titanium dioxide nanoparticles on endothelial cells by the intracellular pathway. Environ. Toxicol..

[107] Jan R., Chaudhry G.E. (2019). Understanding Apoptosis and Apoptotic Pathways Targeted Cancer Therapeutics. Adv. Pharm. Bull..

[108] Tait S.W., Green D.R. (2013). Mitochondrial regulation of cell death. Cold Spring Harb. Perspect. Biol..

[109] Chen L., Yokel R.A., Hennig B., Toborek M. (2008). Manufactured aluminum oxide nanoparticles decrease expression of tight junction proteins in brain vasculature. J. Neuroimmune Pharmacol..

[110] Huang F., Feng Y., Wang Z.A., Cao Y., Yan Q., Wang W., Feng S. (2025). Environmentally Relevant Concentrations of Commercial Titanium Dioxide Nanoparticles Induce Ferroptosis in HUVECs. Environ. Toxicol..

[111] Stockwell B.R., Friedmann Angeli J.P., Bayir H., Bush A.I., Conrad M., Dixon S.J., Fulda S., Gascon S., Hatzios S.K., Kagan V.E. (2017). Ferroptosis: A Regulated Cell Death Nexus Linking Metabolism, Redox Biology, and Disease. Cell.

[112] Wu X., Li Y., Zhang S., Zhou X. (2021). Ferroptosis as a novel therapeutic target for cardiovascular disease. Theranostics.

[113] Zhang X., Kong F., Wang T., Huang X., Li W., Zhang M., Wen T., Liu J., Zhang Y., Meng J. (2022). Iron oxide nanoparticles cause surface coating- and core chemistry-dependent endothelial cell ferroptosis. Nanotoxicology.

[114] Qin X., Zhang J., Wang B., Xu G., Yang X., Zou Z., Yu C. (2021). Ferritinophagy is involved in the zinc oxide nanoparticles-induced ferroptosis of vascular endothelial cells. Autophagy.

[115] Pizzino G., Irrera N., Cucinotta M., Pallio G., Mannino F., Arcoraci V., Squadrito F., Altavilla D., Bitto A. (2017). Oxidative Stress: Harms and Benefits for Human Health. Oxid. Med. Cell Longev..

[116] Tekin S., Seven E. (2022). Assessment of serum catalase, reduced glutathione, and superoxide dismutase activities and malondialdehyde levels in keratoconus patients. Eye.

[117] Jena A.B., Samal R.R., Bhol N.K., Duttaroy A.K. (2023). Cellular Red-Ox system in health and disease: The latest update. Biomed. Pharmacother..

[118] Panda P., Verma H.K., Lakkakula S., Merchant N., Kadir F., Rahman S., Jeffree M.S., Lakkakula B., Rao P.V. (2022). Biomarkers of Oxidative Stress Tethered to Cardiovascular Diseases. Oxid. Med. Cell Longev..

[119] Duan D., Li H., Chai S., Zhang L., Fan T., Hu Z., Feng Y. (2024). The relationship between cardiac oxidative stress, inflammatory cytokine response, cardiac pump function, and prognosis post-myocardial infarction. Sci. Rep..

[120] Aimo A., Castiglione V., Borrelli C., Saccaro L.F., Franzini M., Masi S., Emdin M., Giannoni A. (2020). Oxidative stress and inflammation in the evolution of heart failure: From pathophysiology to therapeutic strategies. Eur. J. Prev. Cardiol..

[121] de Vries D.K., Kortekaas K.A., Tsikas D., Wijermars L.G., van Noorden C.J., Suchy M.T., Cobbaert C.M., Klautz R.J., Schaapherder A.F., Lindeman J.H. (2013). Oxidative damage in clinical ischemia/reperfusion injury: A reappraisal. Antioxid. Redox. Signal..

[122] Chen I.C., Hsiao I.L., Lin H.C., Wu C.H., Chuang C.Y., Huang Y.J. (2016). Influence of silver and titanium dioxide nanoparticles on in vitro blood-brain barrier permeability. Environ. Toxicol. Pharmacol..

[123] Jang J., Park S., Choi I.H. (2021). Increased Interleukin-11 and Stress-Related Gene Expression in Human Endothelial and Bronchial Epithelial Cells Exposed to Silver Nanoparticles. Biomolecules.

[124] Siddiqui M.A., Wahab R., Saquib Q., Ahmad J., Farshori N.N., Al-Sheddi E.S., Al-Oqail M.M., Al-Massarani S.M., Al-Khedhairy A.A. (2023). Iron oxide nanoparticles induced cytotoxicity, oxidative stress, cell cycle arrest, and DNA damage in human umbilical vein endothelial cells. J. Trace Elem. Med. Biol..

[125] Shi J., Sun X., Lin Y., Zou X., Li Z., Liao Y., Du M., Zhang H. (2014). Endothelial cell injury and dysfunction induced by silver nanoparticles through oxidative stress via IKK/NF-kappaB pathways. Biomaterials.

[126] Gholinejad Z., Khadem Ansari M.H., Rasmi Y. (2019). Titanium dioxide nanoparticles induce endothelial cell apoptosis via cell membrane oxidative damage and p38, PI3K/Akt, NF-kappaB signaling pathways modulation. J. Trace. Elem. Med. Biol..

[127] Stevens T., Garcia J.G., Shasby D.M., Bhattacharya J., Malik A.B. (2000). Mechanisms regulating endothelial cell barrier function. Am. J. Physiol. Lung. Cell Mol. Physiol..

[128] Claesson-Welsh L., Dejana E., McDonald D.M. (2021). Permeability of the Endothelial Barrier: Identifying and Reconciling Controversies. Trends Mol. Med..

[129] Medina-Leyte D.J., Zepeda-Garcia O., Dominguez-Perez M., Gonzalez-Garrido A., Villarreal-Molina T., Jacobo-Albavera L. (2021). Endothelial Dysfunction, Inflammation and Coronary Artery Disease: Potential Biomarkers and Promising Therapeutical Approaches. Int. J. Mol. Sci..

[130] Wu D., Chen Q., Chen X., Han F., Chen Z., Wang Y. (2023). The blood-brain barrier: Structure, regulation, and drug delivery. Signal. Transduct. Target Ther..

[131] Chen T., Dai Y., Hu C., Lin Z., Wang S., Yang J., Zeng L., Li S., Li W. (2024). Cellular and molecular mechanisms of the blood-brain barrier dysfunction in neurodegenerative diseases. Fluids Barriers CNS.

[132] Xu L., Dan M., Shao A., Cheng X., Zhang C., Yokel R.A., Takemura T., Hanagata N., Niwa M., Watanabe D. (2015). Silver nanoparticles induce tight junction disruption and astrocyte neurotoxicity in a rat blood-brain barrier primary triple coculture model. Int. J. Nanomed..

[133] Chan Y.J., Liao P.L., Tsai C.H., Cheng Y.W., Lin F.L., Ho J.D., Chen C.Y., Li C.H. (2021). Titanium dioxide nanoparticles impair the inner blood-retinal barrier and retinal electrophysiology through rapid ADAM17 activation and claudin-5 degradation. Part. Fibre Toxicol..

[134] Zhang X., Lin Y., Xin J., Zhang Y., Yang K., Luo Y., Wang B. (2024). Red blood cells in biology and translational medicine: Natural vehicle inspires new biomedical applications. Theranostics.

[135] Zhou Z. (2021). Purinergic interplay between erythrocytes and platelets in diabetes-associated vascular dysfunction. Purinergic. Signal..

[136] Byrnes J.R., Wolberg A.S. (2017). Red blood cells in thrombosis. Blood.

[137] Pernow J., Mahdi A., Yang J., Zhou Z. (2019). Red blood cell dysfunction: A new player in cardiovascular disease. Cardiovasc. Res..

[138] Sun J., Vyas P., Mann S., Paganini-Hill A., Nunes A.C.F., Lau W.L., Cribbs D.H., Fisher M.J., Sumbria R.K. (2021). Insights Into the Mechanisms of Brain Endothelial Erythrophagocytosis. Front. Cell Dev. Biol..

[139] Zlamal J., Singh A., Weich K., Jaffal H., Uzun G., Pelzl L., Althaus K., Bakchoul T. (2023). Platelet phosphatidylserine is the critical mediator of thrombosis in heparin-induced thrombocytopenia. Haematologica.

[140] Wang L., Bi Y., Yu M., Li T., Tong D., Yang X., Zhang C., Guo L., Wang C., Kou Y. (2018). Phosphatidylserine-exposing blood cells and microparticles induce procoagulant activity in non-valvular atrial fibrillation. Int. J. Cardiol..

[141] Kim E.H., Baek S.M., Choi S., Cho J., Tahmasebi S., Bae O.N. (2024). Promoted coagulant activity and disrupted blood-brain barrier depending on phosphatidylserine externalization of red blood cells exposed to ZnO nanoparticles. Environ. Pollut.

[142] Bian Y., Kim K., Ngo T., Kim I., Bae O.N., Lim K.M., Chung J.H. (2019). Silver nanoparticles promote procoagulant activity of red blood cells: A potential risk of thrombosis in susceptible population. Part. Fibre Toxicol..

[143] Bian Y., Chung H.Y., Bae O.N., Lim K.M., Chung J.H., Pi J. (2021). Titanium dioxide nanoparticles enhance thrombosis through triggering the phosphatidylserine exposure and procoagulant activation of red blood cells. Part. Fibre Toxicol..

[144] Henein M.Y., Vancheri S., Longo G., Vancheri F. (2022). The Role of Inflammation in Cardiovascular Disease. Int. J. Mol. Sci..

[145] Gerogianni A., Bal M., Mohlin C., Woodruff T.M., Lambris J.D., Mollnes T.E., Sjostrom D.J., Nilsson P.H. (2023). In vitro evaluation of iron oxide nanoparticle-induced thromboinflammatory response using a combined human whole blood and endothelial cell model. Front. Immunol..

[146] Park E.J., Kim S.N., Yoon C., Cho J.W., Lee G.H., Kim D.W., Park J., Choi I., Lee S.H., Song J. (2021). Repeated intratracheal instillation of zinc oxide nanoparticles induced pulmonary damage and a systemic inflammatory response in cynomolgus monkeys. Nanotoxicology.

[147] Monse C., Hagemeyer O., Raulf M., Jettkant B., van Kampen V., Kendzia B., Gering V., Kappert G., Weiss T., Ulrich N. (2018). Concentration-dependent systemic response after inhalation of nano-sized zinc oxide particles in human volunteers. Part. Fibre Toxicol..

[148] Ajoolabady A., Pratico D., Lin L., Mantzoros C.S., Bahijri S., Tuomilehto J., Ren J. (2024). Inflammation in atherosclerosis: Pathophysiology and mechanisms. Cell Death Dis..

[149] Zhu M.T., Wang B., Wang Y., Yuan L., Wang H.J., Wang M., Ouyang H., Chai Z.F., Feng W.Y., Zhao Y.L. (2011). Endothelial dysfunction and inflammation induced by iron oxide nanoparticle exposure: Risk factors for early atherosclerosis. Toxicol. Lett..

[150] Gojova A., Guo B., Kota R.S., Rutledge J.C., Kennedy I.M., Barakat A.I. (2007). Induction of inflammation in vascular endothelial cells by metal oxide nanoparticles: Effect of particle composition. Environ. Health Perspect.

[151] Gojova A., Lee J.T., Jung H.S., Guo B., Barakat A.I., Kennedy I.M. (2009). Effect of cerium oxide nanoparticles on inflammation in vascular endothelial cells. Inhal. Toxicol..

[152] Yousef M.I., Abuzreda A.A., Kamel M.A. (2019). Cardiotoxicity and lung toxicity in male rats induced by long-term exposure to iron oxide and silver nanoparticles. Exp. Ther. Med..

[153] Wu L., Wen W., Wang X., Huang D., Cao J., Qi X., Shen S. (2022). Ultrasmall iron oxide nanoparticles cause significant toxicity by specifically inducing acute oxidative stress to multiple organs. Part. Fibre. Toxicol..

[154] Avsievich T., Popov A., Bykov A., Meglinski I. (2019). Mutual interaction of red blood cells influenced by nanoparticles. Sci. Rep..

[155] Magaye R.R., Yue X., Zou B., Shi H., Yu H., Liu K., Lin X., Xu J., Yang C., Wu A. (2014). Acute toxicity of nickel nanoparticles in rats after intravenous injection. Int. J. Nanomed..

[156] Chen Z., Wang Y., Zhuo L., Chen S., Zhao L., Luan X., Wang H., Jia G. (2015). Effect of titanium dioxide nanoparticles on the cardiovascular system after oral administration. Toxicol. Lett..

[157] Odzak N., Kistler D., Behra R., Sigg L. (2014). Dissolution of metal and metal oxide nanoparticles in aqueous media. Environ. Pollut.

[158] Zhang H., Ji Z., Xia T., Meng H., Low-Kam C., Liu R., Pokhrel S., Lin S., Wang X., Liao Y.P. (2012). Use of metal oxide nanoparticle band gap to develop a predictive paradigm for oxidative stress and acute pulmonary inflammation. ACS Nano.

[159] Wehmas L.C., Anders C., Chess J., Punnoose A., Pereira C.B., Greenwood J.A., Tanguay R.L. (2015). Comparative Metal Oxide Nanoparticle Toxicity Using Embryonic Zebrafish. Toxicol. Rep..

[160] Xiao Y., Vijver M.G., Chen G., Peijnenburg W.J. (2015). Toxicity and accumulation of Cu and ZnO nanoparticles in Daphnia magna. Environ. Sci. Technol..

[161] Yazdanshenas M.R., Rezaei M.R., Kharkan J. (2025). Comparative toxicity of zinc oxide nanoparticles and zinc salts in male mice: Hematological, biochemical, and histopathological impacts. Toxicol. Rep..

[162] Lee I.C., Ko J.W., Park S.H., Lim J.O., Shin I.S., Moon C., Kim S.H., Heo J.D., Kim J.C. (2016). Comparative toxicity and biodistribution of copper nanoparticles and cupric ions in rats. Int. J. Nanomed..

[163] Boudreau M.D., Imam M.S., Paredes A.M., Bryant M.S., Cunningham C.K., Felton R.P., Jones M.Y., Davis K.J., Olson G.R. (2016). Differential Effects of Silver Nanoparticles and Silver Ions on Tissue Accumulation, Distribution, and Toxicity in the Sprague Dawley Rat Following Daily Oral Gavage Administration for 13 Weeks. Toxicol. Sci..

[164] Khan M., Naqvi A.H., Ahmad M. (2015). Comparative study of the cytotoxic and genotoxic potentials of zinc oxide and titanium dioxide nanoparticles. Toxicol. Rep..

[165] Valdiglesias V., Alba-Gonzalez A., Fernandez-Bertolez N., Touzani A., Ramos-Pan L., Reis A.T., Moreda-Pineiro J., Yanez J., Laffon B., Folgueira M. (2023). Effects of Zinc Oxide Nanoparticle Exposure on Human Glial Cells and Zebrafish Embryos. Int. J. Mol. Sci..

[166] Zhang C., Liu Z., Zhang Y., Ma L., Song E., Song Y. (2020). “Iron free” zinc oxide nanoparticles with ion-leaking properties disrupt intracellular ROS and iron homeostasis to induce ferroptosis. Cell Death Dis..

